# Reliability and validity of a revised version of the General Nutrition Knowledge Questionnaire

**DOI:** 10.1038/ejcn.2016.87

**Published:** 2016-06-01

**Authors:** N Kliemann, J Wardle, F Johnson, H Croker

**Affiliations:** 1 Health Behaviour Research Centre, Department of Epidemiology and Public Health, University College London, London, UK

## Abstract

**Background/Objectives:**

The General Nutrition Knowledge Questionnaire (GNKQ) was developed in the 1990s and has been widely used. Since then advances in understanding of associations between diet and disease have led to changes in dietary recommendations. This study reports the validity and reliability of an updated version of the GNKQ, bringing it into line with current nutritional advice.

**Methods/Subjects:**

Following a review of current recommendations, the revised version of the GNKQ (GNKQ-R) was created, consisting of 88 items and four sections. Reliability and validity of the GNKQ-R were determined in four validation studies: (1) reliability was examined using an online sample (*n*=266), (2) construct validity was assessed with 96 Dietetics students and 89 english students using the ‘known-groups' method, (3) associations between nutrition knowledge and socio-demographic characteristics were examined using the previously described samples and (4) sensitivity to change was tested by measuring GNKQ-R scores pre- and post-exposure to online nutrition information in written (*n*=65) and video (*n*=41) formats.

**Results:**

The reliability was >0.7 in all sections. Dietetics students scored significantly higher than english students. As predicted, GNKQ-R scores were significantly higher among females vs males, people with a degree vs without, and people with very good vs poor or good health status. They were lower in those older than 50 years vs younger adults. GNKQ-R scores were significantly greater after the nutrition interventions in both written and video formats.

**Conclusions:**

The GNKQ-R is a valid measure of nutrition knowledge that is consistent, reliable and sensitive to change.

## Introduction

The General Nutrition Knowledge Questionnaire (GNKQ) has been widely used by the scientific community. It was developed and validated for the UK adult population in the 1990s by Parmenter and Wardle^1^ and has also been adapted and validated for other populations.^2, 3, 4, 5^ Research using the GNKQ has found that nutrition knowledge is associated with demographic characteristics such as gender, age, level of education and socio-economic status.^6, 7^ Studies have also found a positive and significant relationship between the GNKQ score and healthy dietary intake when adjusted for demographic characteristics^8, 9^ but no relationship with weight status.^10^

However, since the GNKQ was developed, advances in understanding of the associations between diet and health have led to changes in advice regarding good nutrition. Developments in the food supply, including the introduction of new types of foods and processing methods, have also resulted in frequent updates to nutrition recommendations,^11, 12^ and these developments necessitate a periodic re-evaluation of measures designed to assess nutrition knowledge.^13, 14^ Some aspects of nutrition now known to be associated with health outcomes, such as omega-3 fatty acids, trans-fats and refined carbohydrate consumption,^11, 15, 16^ are not referenced in the original GNKQ. Indeed, this has already been recognised by researchers who have modified and validated the original GNKQ for other populations; for example, the Turkish version of the GNKQ has added items on trans-fat sources.^2^

In failing to cover all aspects of current nutritional knowledge, or by using questions that are outdated, the GNKQ is unlikely to be as valid a measure of nutrition knowledge today as it was at the time of its development. This study aimed to bring the GNKQ up-to-date with current recommendations and to establish the validity and reliability of the revised scale.

## Methods

### Development of the GNKQ-R

Current UK and international nutritional guidelines were collated and reviewed for content. Discrepancies between these recommendations and the items from the original GNKQ were identified. Documents consulted included reports from Public Health England,^17, 18^ UK Scientific Advisory Committee on Nutrition,^19^ World Cancer Research Fund,^20^ World Health Organization,^15, 16, 21, 22, 23^ and other academic and public-health sources of nutrition information.^9, 11, 24, 25, 26, 27, 28, 29, 30^

Informed by this review, a revised version of the GNKQ was designed as a structured scale initially with 97 items. An expert panel composed of 2 dieticians, 3 health psychologists and 15 dietetics students assessed the level of difficulty and adequacy of the revised nutrition knowledge questionnaire and selected the best items in terms of clarity, content and interpretability. This resulted in the removal of another 9 items.

### Assessing reliability and validity of the GNKQ-R

The psychometric properties of the GNKQ-R were tested in four studies that examined the scale's internal and external reliability (study 1), construct validity (study 2), convergent validity (study 3) and sensitivity to change in nutrition knowledge (responsiveness of scores to nutrition information; study 4). In all cases, data were collected using online surveys.

#### Study 1: Assessment of internal and external reliability

Participants: Participants for study 1 were recruited from two different sources, in order to achieve a similar sample to the UK population in terms of gender, educational level and weight status percentages.^31^ A stratified sample of 185 participants aged 18 years or older was recruited using Research Now, an online market research company, which has access to a panel of over 6 000 000 UK residents. Stratification variables were gender (50% male) and educational level (75% people without a degree). Panellists were randomly selected and invited by email to take part in the research. An e-Rewards (points) incentive was offered for participation. These points, when accumulated, can be redeemed for a variety of rewards. According to a systematic review, incentives may increase the response rate of online health surveys.^32^ A second group of 81 participants was recruited through Weight Concern, a charity addressing the needs of overweight people. Weight Concern manages an online panel (The Big Panel) composed of around 1850 UK residents who have first-hand experience of being overweight, and all members were invited to participate via email. No incentives were offered.

Design and procedure: Both groups completed the GNKQ-R online, and data from the samples were combined. The completion of the online questionnaire should take no longer than 15 min, and the time spent by each individual was recorded. The internal reliability of the scale (whether the items in the questionnaire assess nutrition knowledge consistently) and its external (test–retest) reliability were assessed.^33^ Internal reliability was determined by corrected item–total correlation, which should be ⩾0.2,^34, 35^ and Cronbach's alpha for the overall score and for each section, which should be ⩾0.7.^35^ A test–retest approach was used to examine the reliability of the questionnaire over time. Two weeks after the initial recruitment email was sent, all Research Now participants (*N*=185), and those from the Big Panel who had agreed to be contacted again (*N*=61), were invited to complete the questionnaire for the second time. Recruitment was closed when the required sample size of 100 was reached. Paired *t*-tests and intraclass correlation coefficients were calculated for the overall score and for each section. Minimum requirement for intraclass correlation coefficients is that it should be >0.7.^33^

#### Study 2: Assessment of construct validity

Participants: The sample for study 2 was composed of third-year or postgraduate students studying either nutrition/dietetics (*N*=96) (subsequent references to ‘dietetics courses' refer to both nutrition and dietetics courses) or english (*N*=89) at UK universities. Course leaders at all of the 14 universities who offer dietetics courses in the UK were contacted. Leaders of 12 english courses identified in the same universities and/or same cities as dietetics courses were also invited. A total of nine dietetics courses and six english courses agreed to take part by forwarding the questionnaire to their students. These universities represented eight different regions in the UK: London, Wales, Yorkshire, East Midlands, South West, North West, Northern Ireland and Scotland. All participants were invited to enter a prize draw for a £25 high street voucher.

Design and procedure: Participants were invited to complete the online questionnaire, and the time taken by each individual was recorded. Construct validity was established via the ‘known-groups method', comparing two groups that are expected to differ in their nutrition knowledge (dietetics and english students) but are similar in terms of age, gender and socio-economic status.^1^ Independent *t*-tests were used to compare the scores of the two groups of students for the overall questionnaire and for each section. Cohen's effect sizes were also calculated.

#### Study 3: Assessment of convergent validity

Participants: Study 3 used the data collected from participants in studies 1 and 2 combined (*N*=451).

Design and procedure: Convergent validity was assessed by analysing relationships between the GNKQ-R and education, age, gender and health status. It was expected that nutrition knowledge would increase with education and health status^6, 7, 36, 37^ and would be higher among women.^6, 7, 38^ It was also anticipated that nutrition knowledge would be better among middle-aged adults compared with younger and older adults;^6^ although previous studies have sometimes found other patterns of association between age and knowledge.^7, 10^

For the purpose of the analyses and based on previous results for the original GNKQ,^6^ education was divided into 2 groups and age and health status into three. The associations between nutrition knowledge scores (overall and by section), and gender and education were tested using independent *t*-tests. Associations between nutrition knowledge scores (overall and by section) and age and health status were examined using one-way ANOVAs, with *post hoc* Tukey's tests.

#### Study 4: Change over time: scale sensitivity to nutritional information

Participants: Data for study 4 were collected from two participant samples. Participants were recruited through Research Now (*N*=65) using the same methods as for study 1. A further sample was recruited from students and staff of University College London (*N*=40). An email with information about the study and the link to the questionnaire was sent to 5 academic departments at University College London.

Design and procedure: In study 4, the responsiveness of scale scores to exposure to nutritional information was tested.^34^ Responsiveness to change was evaluated using two uncontrolled before–after study designs, and each of them delivered a nutrition education approach that has shown to be effective at promoting nutrition knowledge in adults.^39, 40, 41, 42^ The first approach used an online written nutrition information intervention. Participants who completed the 15-min online Research Now questionnaire were re-contacted 2 weeks later and invited to complete the questionnaire again while reading 20 on-screen pieces of nutrition information. Similarly to the online questionnaire provided in the previous studies, the GNKQ-R was broken down into many different online pages and after participant clicking on ‘next page' they were not allowed to go back. The nutritional information was placed through different pages of the online questionnaire, being always before, but not on the same page, as the questions related to its content. The reason for that was to not permit participants to go back to reread the nutritional information before answering the question. The nutrition information was taken from publically accessible websites of major public health organisations and charities in the UK, including sites created by the NHS, British Heart Foundation, Cancer Research UK, Weight Concern, British Dietetic Association, British Nutrition Foundation and Diabetes UK. The time participants took responding to the questionnaire was recorded. The second approach used an online educational video intervention to assess responsiveness to the change of the Dietary recommendation section of the GNKQ-R. This intervention was focused only on GNKQ-R's section one because the video covered only the nutrition information assessed in this section. University College London participants completed the 5-min questionnaire, and 2 weeks later they were invited to watch a 7-min long video before completing the questionnaire for the second time. The time spent responding to the questionnaire was recorded. The video displayed was a short version of Healthy Eating by British Nutrition Foundation.^43^ No incentives were given to participants for either intervention. The sample size for each of these groups exceeded the minimum size of *N*=34 participants required to detect a medium effect size (*d*=0.5), with 80% power at the 5% significance level, as assessed using G* Power (version 3.1; Heinrich Heine University Düsseldorf, Germany). Paired *t*-tests were applied to compare the overall score and each section's score before and after the video and online nutrition information interventions. Cohen's effect sizes were also calculated.

### Analysis

All analyses were performed using IBM SPSS statistics version 22 (SPSS Inc., Chicago, IL, USA). Knowledge scores were calculated for each section and for the overall questionnaire; a score of one was given for each correct response and scores summed. Demographic information on gender, age, BMI, health status, marital status, children under 18 years living at home, ethnic origin, level of education and nutrition-related qualification was collected for each sample. The median time spent completing each online questionnaire was assessed.

### Ethical approval

The study received ethical approval by the Ethics Committee of the University College London, UK, and also from the University of Surrey for the content validity study with Dietetics and Nutrition students. All participants gave informed consent, which was obtained when participants clicked on ‘Next page' to start the online survey.

## Results

The GNKQ-R and details of changes made to the original GNKQ are available as Supplementary Information at the journal's website. Initially, 74 items were removed and 36 were kept and adapted. An additional 61 items were created, resulting in a first draft questionnaire of 97 items. Nine items were removed following consultation with the expert panel, on the basis of their clarity, content or interpretability, leaving a final questionnaire of 88 items.

### Reliability and validity of the GNKQ-R

Demographic characteristics of the samples used in each of the four validation studies are shown in Table 1. An imbalance in gender composition was observed in all studies, all being samples composed mainly of women. The time expected to complete the online questionnaire in study 1 and study 2 was 15 min each, and the median time spent to complete each questionnaire was 14 min. In study 4, 15 min was the time expected to complete the online intervention and 4 min the video intervention. The median time spent to complete the online intervention questionnaire was 18 min and the video intervention questionnaire was 4 min. A table with this information about the time expected and spent to complete each questionnaire is available as a Supplementary Material at the journal's website.

#### Study 1: Assessment of internal and external reliability

The reliability analysis resulted in 4 items failing to meet the item discrimination criterion (item–total correlation ⩾0.2). However, all items were retained on the grounds of content validity. The overall internal reliability, shown in Table 2, was high (Cronbach's alpha=0.93), as was the internal reliability of each section (Cronbach's alpha=0.70–0.86). The external reliability outcome was greater than the recommended criteria of 0.7 (intraclass correlation coefficient=0.72–0.89). Paired *t*-tests showed no significant differences between scores at time 1 and 2 for all sections.

#### Study 2: Assessment of construct validity

There was a roughly equal split of students studying dietetics vs english (52 vs 48%) and no significant differences for gender, age, ethnic origin, weight status or education (Table 1). There was a significant difference for self-rated health status, with Dietetics students more likely to rate this as ‘very good'.

Dietetics students scored significantly higher than english students on all sections (Table 3). The overall mean score difference was 11.5 (95% confidence of interval: 9.3; 13.7), which represents a large-sized effect (*d*=1.2).

#### Study 3: Assessment of convergent validity

Associations between nutrition knowledge score and demographic and health-related variables are shown in Table 4. Knowledge (for individual sections and overall score) was significantly higher among women and those with higher education (both having medium to large effect sizes) and lower among those with poor health status (small effect size). Older adults had overall and dietary recommendation scores significantly lower than younger and middle-aged adults; and this represented a small effect size.

#### Study 4: Change over time: scale sensitivity to nutritional information

Nutrition knowledge scores significantly improved after receiving the written information intervention for all sections apart from healthy food choices (Table 5). The differences found between time 1 and 2 represented a large-size effect for dietary recommendations, a medium-sized effect for food groups and a small-sized effect for diet, disease and weight. No significant difference was found between time 1 and 2 for the healthy food choices section.

Forty students and staff at University College London participated in the video intervention. Participants achieved higher scores for dietary recommendations after watching the video (pre- to post-scores: *M*=14.6, SE=0.2 vs *M*=16.2, SE=0.2). This 1.5 point difference (95% confidence of interval: 1.1; 1.9) was significant (*t*(39)=7.5, *P*<0.001) and represented a large-sized effect (*d*=1.1).

## Discussion

The aims of the present study were to update the original GNKQ and to confirm the revised questionnaire's reliability and validity. Revisions to the content were informed by examining current nutrition recommendations, identifying redundant GNKQ items and adding new ones to correspond to current guidelines. The content validity of the revised version was assessed by the research team (composed of dieticians and health psychologists) and by a group of students in dietetics.

The revised questionnaire showed good internal and external reliability, in line with the reliability results of the original GNKQ,^1^ demonstrating that the questionnaire is measuring nutrition knowledge consistently. All knowledge sections had good internal consistency and test–retest reliability. The food groups section was the most reliable section; however, this might be due to the larger number of items, as Cronbach's alpha generally increases as the number of items increases.^35^

As expected, Dietetic students scored significantly higher than english students on all sections, demonstrating that the questionnaire has adequate construct validity (that is, discriminating between groups with higher and lower nutrition knowledge). The previously validated versions of the GNKQ had also shown good construct validity by the ‘known-groups' method.^1, 2, 3, 4^

Expected associations were seen between nutrition knowledge scores and most of the demographic and health status variables, demonstrating good convergent validity. Women and individuals with higher education and self-reported health status scored significantly higher overall and for each section. Older adults obtained the lowest score for each section, in agreement with the original GNKQ results.^6^ However, younger-aged adults had a similar score to middle-aged adults, which may indicate an improvement in access to nutrition information and greater interest in healthy eating by young British adults in 2015 compared with 2000 when the original GNKQ validation study was conducted.^6^

The questionnaire was able to detect changes in nutrition knowledge over time. The online written nutrition information intervention significantly improved knowledge scores overall and for three of the individual sections (dietary recommendations, food groups, and diet, disease and weight associations). The video intervention significantly improved the dietary recommendations score. Further studies could be conducted to test whether providing nutrition skill training would make a significant difference to the healthy food choices knowledge score in adults.

There are some limitations that may affect the generalisability of these results. The findings regarding the validity, reliability and responsiveness to change are limited to the context within which the data were collected. Future studies are needed to test the validity of the questionnaire in different populations (for example, ethnic minorities and other countries). For convenience, university students and staff were invited to take part in the video intervention, and these are unlikely to reflect the educational and socio-economic status of the general population. Moreover, the two interventions used a before–after study design^44^ and did not have a control group. However, as the study did not aim to assess the intervention effectiveness, the design was adequate to establish score improvement following exposure to information. All the data collection was online, which means that those without computer or internet access were excluded. In addition, there is no information about how many people actually received the invitation but chose not to participate in each stage of the study. People with a greater interest in nutrition are more likely to have opted in. Weight and height were self-reported. However, even though direct measurement of weight is usually recommended,^45^ studies have shown that adults, especially young adults, give a valid online self-report weight.^46^ The possibility that participants guessing or searching for the correct response is also a limitation. However, in order to reduce guessing all questions had the option ‘not sure', and the instructions stated clearly that people should mark ‘not sure' rather than guess. To reduce opportunities for participants making corrections, they were not allowed to go back and edit their responses at any point. Mean time spent to complete each online questionnaire was similar to the time expected, suggesting that the search for the right answers was not widespread.

In conclusion, the revised 88-item instrument has been shown to be a measure of nutrition knowledge that is consistent, reliable, valid and sensitive to changes in knowledge. The sections can be administered individually and give valid and reliable results for specific areas of nutrition knowledge. The revised version of the GNKQ is likely to be a useful tool to assess nutrition knowledge among the UK adult population.

## Figures and Tables

**Table 1:** Socio-demographic characteristics of the samples

	*Study 1*		*Study 2*			*Study 3*	*Study 4*	
	*All participants* (n=*266*)	*Retest* (n=*101*)	*Nutrition students* (n=*96*)	*English students* (n=*89*)	*X*^*2*^ (*df*)	*Sample* (n=*451*)	*Online information* (n=*65*)	*Video information* (n=*40*)
	*%* (n)	*%* (n)	*%* (n)	*%* (n)		*%* (n)	*%* (n)	*%* (n)
*Gender*								
Male	36.8 (98)	16.8 (17)	7.3 (7)	12.4 (11)	1.35 (1)**	27.5 (116)	36.9 (24)	25 (10)
Female	63.2 (168)	83.2 (84)	92.7 (89)	87.6 (78)		74.3 (335)	63.1 (41)	75 (30)
Missing	0 (0)	0 (0)	0 (0)	0 (0)		0 (0)	0 (0)	0 (0)

*Age*								
18–35	13.9 (37)	9.9 (10)	84.4 (81)	86.5 (77)	1.92 (2)**	43.2 (195)	10.8 (7)	72.5 (29)
36–50	32 (85)	37.6 (38)	14.6 (14)	10.1 (9)		23.9 (108)	20 (13)	12.5 (5)
>50	53.4 (142)	52.5 (53)	1 (1)	3.4 (3)		32.4 (146)	67.7 (44)	15 (6)
Missing	0.8 (2)	0 (0)	0 (0)	0 (0)		0.4 (2)	0 (0)	0 (0)
								
*Ethnic origin*								
White^a^	94.4 (251)	93.1 (94)	90.6 (87)	79.8 (71)	8.63 (3)**	90.7 (409)	93.8 (61)	67.5 (27)
Black^b^	0.4 (1)	0 (0)	0 (0)	2.2 (2)		0.7 (3)	1.5 (1)	5 (2)
Asian^c^	2.3 (6)	2 (2)	4.2 (4)	14.6 (13)		5.1 (23)	3.1 (2)	17.5 (7)
Mixed^d^	3 (8)	5 (5)	5.2 (5)	3.4 (3)		3.5 (16)	1.5 (1)	10 (4)
Missing	0 (0)	0 (0)	0 (0)	0 (0)		0 (0)	0 (0)	0 (0)
								
*Marital status*								
Single	18 (48)	17.8 (18)	75 (72)	78.7 (70)	2.02 (2)**	42.1 (190)	12.3 (8)	60 (24)
Married	69.5 (185)	68.3 (69)	24 (23)	18 (16)		49.7 (224)	73.8 (48)	40 (16)
Separated/widowed^e^	12.4 (33)	13.9 (14)	1 (1)	3.4 (3)		8.2 (37)	13.8 (9)	0 (0)
Missing	0 (0)	0 (0)	0 (0)	0 (0)		0 (0)	0 (0)	0 (0)
								
*Have children <18*								
Yes	22.6 (60)	27.7 (28)	14.6 (14)	10.1 (9)	0.84 (1)**	18.4 (83)	21.5 (14)	15 (6)
No	77.1 (205)	71.3 (72)	84.4 (81)	88.8 (79)		80.9 (365)	78.5 (51)	85 (34)
Missing	0.4 (1)	1 (1)	1 (1)	1.1 (1)		0.7 (3)	0 (0)	0 (0)

*Education*								
Secondary school	7.9 (21)	7.9 (8)	1 (1)	1.1 (1)	4.07 (3)**	5.1 (23)	13.8 (9)	0 (0)
O levels to A levels	29.7 (79)	30.7 (31)	26 (25)	36 (32)		30.2 (136)	40 (26)	20 (8)
Certificate/diploma	26.7 (71)	18.8 (19)	7.3 (7)	2.2 (2)		17.7 (80)	30.8 (20)	5 (2)
Degree/PG degree^f^	35.7 (95)	42.6 (43)	65.6 (63)	60.7 (54)		47 (212)	15.4 (10)	75 (30)
Missing	0 (0)	0 (0)	0 (0)	0 (0)		0 (0)	0 (0)	0 (0)

*Nutrition qualification*								
Yes	15 (40)	18.8 (19)	100 (96)	0 (0)	—	30.2 (136)	10.8 (7)	22.5 (9)
No	85 (226)	81.2 (82)	0 (0)	100 (89)		315 (69.8)	89.2 (58)	77.5 (31)
Missing	0 (0)	0 (0)	0 (0)	0 (0)		0 (0)	0 (0)	0 (0)

*Health status*								
Poor/fair	45.5 (121)	49.5 (50)	7.3 (7)	22.5 (20)	11.5 (2)*	32.8 (148)	33.8 (22)	7.5 (3)
Good	36.5 (97)	33.7 (34)	44.8 (43)	48.3 (43)		40.6 (183)	44.6 (29)	55 (22)
Very good/excellent	18 (48)	16.8 (17)	47.9 (46)	29.2 (26)		26.6 (120)	21.5 (14)	37.5 (15)
Missing	0 (0)	0 (0)	0 (0)	0 (0)		0 (0)	0 (0)	0 (0)

*Weight status*								
Underweight	2.3 (6)	1 (1)	6.3 (6)	6.7 (6)	1.77 (3)	4 (18)	3.1 (2)	7.5 (3)
Normal weight	31.6 (84)	21.8 (22)	77.1 (74)	69.7 (62)		48.8 (220)	52.3 (34)	77.5 (31)
Overweight	28.2 (75)	24.8 (25)	13.5 (13)	18 (16)		23.1 (104)	27.7 (18)	12.5 (5)
Obese	37.2 (99)	52.5 (53)	2.1 (2)	4.5 (4)		23.3 (105)	15.4 (10)	12.5 (5)
Missing	0.8 (2)	0 (0)	1 (1)	1.1 (1)		0.9 (4)	1.5 (1)	2.5 (1)

a White British, White Irish or other White background.

b Black British, Black Caribbean, Black African or other Black background.

c Indian, Pakistani, Bangladeshi, Chinese or other Asian background.

d White and Black Caribbean, White and Black African, White and Asian or other mixed background.

e Separated, divorced or widowed.

f Degree or postgraduate degree.

**P>0.05, *P<0.05.

**Table 2:** Internal reliability and external reliability of the GNKQ-R (study 1)

*Reliability*	*Knowledge section (max possible score)*				
	*Overall (88)*	*Section 1 (18)*	*Section 2 (36)*	*Section 3 (13)*	*Section 4 (21)*
*Internal reliability (*n=*266)*					
Cronbach's alpha	0.93	0.70	0.86	0.72	0.77
*External reliability (*n=*101)*					
Time 1^a^ (mean (SE))	69.3 (1.2)	14.3 (0.2)	27.7 (0.6)	10.8 (0.2)	16.4 (0.3)
Time 2^a^ (mean (SE))	70 (1.2)	14.6 (0.2)	28 (0.6)	10.8 (0.2)	16.5 (0.3)
*T*-test (*P*-value)	−1.10 (100)	−1.40 (100)	−0.73 (100)	−0.24 (100)	−0.47 (100)
ICC^b^	0.89	0.75	0.83	0.72	0.82

a Difference between time 1 and time 2 is 2 weeks.

b Intraclass correlation coefficient.

*P*>0.05. Section 1: dietary recommendation; Section 2: food groups; Section 3: healthy food choices; Section 4: diet, disease and weight associations.

**Table 3:** Differences in nutrition knowledge score between nutrition and english students (study 2)

*Construct validity*	*Knowledge section (max possible score)*				
	*Overall (88)*	*Section 1 (18)*	*Section 2 (36)*	*Section 3 (13)*	*Section 4 (21)*
Nutrition students (mean (SE))	79.3 (0.51)	16.3 (0.14)	31.9 (0.27)	11.7 (0.14)	19.3 (0.15)
English students (mean (SE))	67.7 (0.97)	13.9 (0.25)	27.2 (0.49)	10.7 (0.20)	15.9 (0.28)
*T*-test (df)	10.4 (135)**	8.2 (142)**	8.3 (139)**	4.1 (160)**	10.4 (136)**
Effect size^a^	1.2	1	1	0.5	1.2

a Cohen's effect size.

**P<0.001.

Section 1: Dietary recommendation; Section 2: food groups; Section 3: healthy food choices; Section 4: diet, disease and weight associations.

**Table 4:** Associations between nutrition knowledge score, demographics and health status (study 3)

*Variables*	*Knowledge section (max possible score)*				
	*Overall (88)*	*Section 1 (18)*	*Section 2 (36)*	*Section 3 (13)*	*Section 4 (21)*
*Gender*					
Male (mean (SE))	61.7 (1.1)	13.4 (0.2)	24.2 (0.5)	9.2 (0.2)	14.8 (0.3)
Female (mean (SE))	71.4 (0.6)	14.6 (0.1)	28.5 (0.2)	11.1 (0.1)	17.1 (0.1)
*T*-test (df)	−7.8 (449)**	−4.7 (449)**	−7.3 (449)**	−7 (164)**	−5.6 (167)**
Effect size^a^	0.9	0.5	0.8	1	0.8
					
*Education*					
No degree^b^ (mean (SE))	65.8 (0.8)	13.8 (0.1)	25.9 (0.4)	10.1 (0.1)	15.8 (0.2)
Degree or higher^c^ (mean (SE))	72.3 (0.7)	14.9 (0.1)	29.1 (0.3)	11.1 (0.1)	17.2 (0.2)
*T*-test (df)	−5.8 (447)**	−4.2 (449)**	−6.1 (442)**	−4.5 (445)**	−4.2 (449)**
Effect size^a^	0.6	0.5	0.7	0.5	0.5
					
*Age*					
18–35 years (mean (SE))	70.3 (12.8)	14.7 (2.6)	27.9 (5.9)	10.7 (2.3)	16.8 (3.7)
36–50 years (mean (SE))	69.8 (11.8)	14.2 (2.7)	28 (5.5)	10.8 (2.1)	16.5 (3.1)
>50 years (mean (SE))	66.7 (11.3)^b^	13.9 (2.3)^d^	26.4 (5.7)	10.2 (2.1)	16 (3.1)
F-ratio (*d*, *d*)	3.7 (2, 446)*	4.3 (2, 446)**	3.4 (2, 446)*	2.7 (2, 446)	2.1 (2, 446)
Effect size^a^	0.1	0.1	0.1	0.1	0
					
*Health status*					
Poor (mean (SE))	65.1 (12)	13.7 (2.8)^e^	25.8 (6.2)^e^	10 (2.4)	15.5 (3.2)
Good (mean (SE))	69.1 (12.1)	14.4 (2.4)	27.7 (5.6)	10.6 (2.2)	16.4 (3.5)
Excellent (mean (SE))	73.21 (12.2)	15 (2.2)	29 (5)	11.3 (1.7)	17.7 (2.9)
F-ratio (*d*, *d*)	15.3 (2, 448)**^f^	8.7 (2, 448)**	10.8 (2, 448)**	12.8 (2, 288)**^f^	14.3 (2, 448)**^f^
Effect size^a^	0.2	0.1	0.2	0.2	0.2

a Cohen's effect size.

b Secondary school, O levels to A levels or certificate/diploma.

c Degree or postgraduate degree.

d *Post hoc* Tukey's HSD tests showed that people over 50 years of age had significantly lower nutrition knowledge scores than the other two groups at the 0.05 level of significance.

e *Post hoc* Tukey's HSD tests showed that people who reported poor health status had significantly lower nutrition knowledge scores than the other two groups at the 0.05 level of significance.

f Significant difference between all groups.

**P<0.001, *P<0.05.

Section 1: dietary recommendation; Section 2: food groups; Section 3: healthy food choices; Section 4: diet, disease and weight association.

**Table 5:** Differences in nutrition knowledge before and after the online nutrition information interventions (study 4)

*Nutrition information intervention*	*Knowledge section (max possible score)*				
	*Overall (88)*	*Section 1 (18)*	*Section 2 (36)*	*Section 3 (13)*	*Section 4 (21)*
Time 1^a^ (mean (SE))	63.0 (1.3)	13.2 (0.25)	24.6 (0.6)	9.6 (0.3)	15.5 (0.3)
Time 2^b^ (mean (SE))	67.0 (1.2)	14.6 (0.29)	26.4 (0.5)	9.8 (0.3)	16.1 (0.3)
Paired *t*-test (df)	5.2 (64)**	4.7 (64)**	4.4 (64)**	0.74 (64)	2.2 (64)*
Effect size^c^	0.4	0.7	0.4	0.1	0.2

a Time 1=pre-intervention.

b Time 2=post-intervention (2 weeks later).

c Cohen's effect size.

Section 1: dietary recommendation; Section 2: food groups; Section 3: healthy food choices; Section 4: diet, disease and weight association.
