# Supplementary material for: Reliability and validity of a revised version of the General Nutrition Knowledge Questionnaire
Source: Eur J Clin Nutr. 2016 Jun 1;70(10):1174–80. doi: 10.1038/ejcn.2016.87 (PMC5014128; doi:10.1038/ejcn.2016.87)
Supplement: Supplementary Table 1 [file ejcn201687x1.docx]

| **GENERAL NUTRITION KNOWLEDGE QUESTIONNAIRE** | | | | | | | | | | |
| --- | --- | --- | --- | --- | --- | --- | --- | --- | --- | --- |
|  | | | | | | | | | | |
| \| **This is a survey, not a test. Your answers will help identify which dietary advice people find confusing. It is important that you complete it by yourself. Your answers will remain anonymous. If you don’t know the answer, mark “not sure” rather than guess.**  **Thank you for your time.** \| \| --- \| | | | | | | | | | | |
|  | | | | | | | | | | |
| **Section 1: The first few items are about what advice you think experts are giving us.** | | | | | | | | | | |
|  | | | | | | | | | | |
| 1. | Do health experts recommend that people should be eating more, the same amount, or less of the following foods? (tick one box per food) | | | | | | | | | |
|  | | More | | Same | | | Less | | Not Sure |  |
| Fruit | | ✓ | | □ | | | □ | | □ |  |
| Food and drinks with added sugar | | □ | | □ | | | ✓ | | □ |  |
| Vegetables | | ✓ | | □ | | | □ | | □ |  |
| Fatty foods | | □ | | □ | | | ✓ | | □ |  |
| Processed red meat | | □ | | □ | | | ✓ | | □ |  |
| Wholegrains | | ✓ | | □ | | | □ | | □ |  |
| Salty foods | | □ | | □ | | | ✓ | | □ |  |
| Water | | ✓ | | □ | | | □ | | □ |  |
|  | | | | | | | | | | |
| 2. | How many servings of fruit and vegetables per day do experts advise people to eat as a minimum? (One serving could be, for example, an apple or a handful of chopped carrots)  (tick one) | | | | | | | | | |
| 2 | | □ | | |  | | | | | |
| 3 | | □ | | |  | | | | | |
| 4 | | □ | | |  | | | | | |
| 5 or more | | ✓ | | |  | | | | | |
| Not sure | | □ | | |  | | | | | |
|  | | | | | | | | | | |
| 3. | Which of these types of fats do experts recommend that people should eat less of?  (tick one box per food) | | | | | | | | | |
|  | | Eat less | Not eat less | | | Not sure | |  | | |
| Unsaturated fats | | □ | ✓ | | | □ | |  | | |
| Trans fats | | ✓ | □ | | | □ | |  | | |
| Saturated fats | | ✓ | □ | | | □ | |  | | |
|  | | | | | | | | | | |
| 4. | Which type of dairy foods do experts say people should drink? (tick one) | | | | | | | | | |
| Full fat (e.g. full fat milk) | | □ |  | | | | | | | |
| Reduced fat (e.g. skimmed and semi-skimmed milk) | | ✓ |  | | | | | | | |
| Mixture of full fat and reduced fat | | □ |  | | | | | | | |
| Neither, dairy foods should be avoided | | □ |  | | | | | | | |
| Not sure | | □ |  | | | | | | | |

|  | | | |
| --- | --- | --- | --- |
| 5. | How many times per week do experts recommend that people eat oily fish (e.g. salmon and mackerel)? (tick one) | | |
| 1-2 times per week | | ✓ |  |
| 3-4 times per week | | □ |  |
| Every day | | □ |  |
| Not sure | | □ |  |
|  | | | |
| 6. | Approximately how many alcoholic drinks is the maximum recommended per day (The exact number depends on the size and strength of the drink)? (tick one) | | |
| 1 drink each for men and women | | ✓ |  |
| 2 drinks each for men and women | | □ |  |
| 2 drinks for men and 1 drink for women | | □ |  |
| 3 drinks for men and 2 drinks for women | | □ |  |
| Not sure | | □ |  |
|  | | | |
| 7. | How many times per week do experts recommend that people eat breakfast? (tick one) | | |
| 3 times per week | | □ |  |
| 4 times per week | | □ |  |
| Every day | | ✓ |  |
| Not sure | | □ |  |
|  | | | |
| 8. | If a person has two glasses of fruit juice in a day, how many of their daily fruit and vegetable servings would this count as? (tick one) | | |
| None | | □ |  |
| One serving | | ✓ |  |
| Two servings | | □ |  |
| Three servings | | □ |  |
| Not sure | | □ |  |
|  | | | |
| 9. | According to the ‘eatwell guide’ (a guideline showing the proportions of food types people should eat to have a balanced and healthy diet), how much of a person’s diet should be made up of starchy foods? (tick one) | | |
| Quarter | | □ |  |
| Third | | ✓ |  |
| Half | | □ |  |
| Not sure | | □ |  |

| **Section 2: Experts classify foods into groups. We are interested to see whether people are aware of food groups and the nutrients they contain.** | | | | | | | | | | | | | | |
| --- | --- | --- | --- | --- | --- | --- | --- | --- | --- | --- | --- | --- | --- | --- |
|  |  | | | | | | | | | | | | | |
| 1. | Do you think these foods and drinks are typically high or low in added sugar?  (tick one box per food) | | | | | | | | | | | | | |
|  | | | | | | High in added sugar | | Low in added sugar | | | Not sure | |  | |
| Diet cola drinks | | | | | | □ | | ✓ | | | □ | |  | |
| Natural yoghurt | | | | | | □ | | ✓ | | | □ | |  | |
| Ice cream | | | | | | ✓ | | □ | | | □ | |  | |
| Tomato ketchup | | | | | | ✓ | | □ | | | □ | |  | |
| Melon | | | | | | □ | | ✓ | | | □ | |  | |
|  | | | | | | | | | | | | | | |
| 2. | | | | Do you think these foods are typically high or low in salt? (tick one box per food) | | | | | | | | | | |
|  | | | | | | High in salt | | Low in  salt | | | Not Sure | |  | |
| Breakfast cereals | | | | | | ✓ | | □ | | | □ | |  | |
| Frozen vegetables | | | | | | □ | | ✓ | | | □ | |  | |
| Bread | | | | | | ✓ | | □ | | | □ | |  | |
| Baked beans | | | | | | ✓ | | □ | | | □ | |  | |
| Red meat | | | | | | □ | | ✓ | | | □ | |  | |
| Canned soup | | | | | | ✓ | | □ | | | □ | |  | |
|  | | | | | | | | | | | | | | |
| 3. | | | | Do you think these foods are typically high or low in fibre? (tick one box per food) | | | | | | | | | | |
|  | | | | | | High in fibre | | Low in fibre | | Not Sure | | |  | |
| Oats | | | | | | ✓ | | □ | | □ | | |  | |
| Bananas | | | | | | ✓ | | □ | | □ | | |  | |
| White rice | | | | | | □ | | ✓ | | □ | | |  | |
| Eggs | | | | | | □ | | ✓ | | □ | | |  | |
| Potatoes with skin | | | | | | ✓ | | □ | | □ | | |  | |
| Pasta | | | | | | □ | | ✓ | | □ | | |  | |
|  | | | | | | | | | | | | | | |
| 4. | | | | Do you think these foods are a good source of protein? (tick one box per food) | | | | | | | | | | |
|  | | | | | | Good source of protein | | Not a good source of protein | | | Not sure | |  | |
| Poultry | | | | | | ✓ | | □ | | | □ | |  | |
| Cheese | | | | | | ✓ | | □ | | | □ | |  | |
| Fruit | | | | | | □ | | ✓ | | | □ | |  | |
| Baked beans | | | | | | ✓ | | □ | | | □ | |  | |
| Butter | | | | | | □ | | ✓ | | | □ | |  | |
| Nuts | | | | | | ✓ | | □ | | | □ | |  | |
| 5. | | | Which of the following foods do experts count as starchy foods? (tick one box per food) | | | | | | | | | | | |
|  | | | | | | | Starchy food | Not a starchy food | | | Not sure | |  | |
| Cheese | | | | | | | □ | ✓ | | | □ | |  | |
| Pasta | | | | | | | ✓ | □ | | | □ | |  | |
| Potatoes | | | | | | | ✓ | □ | | | □ | |  | |
| Nuts | | | | | | | □ | ✓ | | | □ | |  | |
| Plantains | | | | | | | ✓ | □ | | | □ | |  | |
|  | |  | | | | | | | | | | | | |
| 6. | | Which is the main type of fat present in each of these foods? (tick one box per food) | | | | | | | | | | | | |
|  | | | | | Polyunsaturated fat | Monounsat-urated fat | | | Saturated fat | | | Cholesterol | | Not sure |
| Olive oil | | | | | □ | ✓ | | | □ | | | □ | | □ |
| Butter | | | | | □ | □ | | | ✓ | | | □ | | □ |
| Sunflower oil | | | | | ✓ | □ | | | □ | | | □ | | □ |
| Eggs | | | | | □ | □ | | | □ | | | ✓ | | □ |
|  | | | | | | | | | | | | | | |
| 7. | | | Which of these foods has the most trans-fat? (tick one) | | | | | | | | | | | |
| Biscuits, cakes and pastries | | | | | | ✓ | |  | | | | | | |
| Fish | | | | | | □ | |  | | | | | | |
| Rapeseed oil | | | | | | □ | |  | | | | | | |
| Eggs | | | | | | □ | |  | | | | | | |
| Not sure | | | | | | □ | |  | | | | | | |
|  | | | | | | | | | | | | | | |
| 8. | | | The amount of calcium in a glass of whole milk compared to a glass of skimmed milk is:  (tick one) | | | | | | | | | | | |
| About the same | | | | | | ✓ | |  | | | | | | |
| Much higher | | | | | | □ | |  | | | | | | |
| Much lower | | | | | | □ | |  | | | | | | |
| Not sure | | | | | | □ | |  | | | | | | |
|  | | | | | | | | | | | | | | |
| 9. | | | Which one of the following nutrients has the most calories for the same weight of food? (tick one) | | | | | | | | | | | |
| Sugar | | | | | | □ | |  | | | | | | |
| Starchy | | | | | | □ | |  | | | | | | |
| Fibre/roughage | | | | | | □ | |  | | | | | | |
| Fat | | | | | | ✓ | |  | | | | | | |
| Not sure | | | | | | □ | |  | | | | | | |
|  | | | | | | | | | | | | | | |
| 10. | | | Compared to minimally processed foods, processed foods are: (tick one) | | | | | | | | | | | |
| Higher in calories | | | | | | ✓ | |  | | | | | | |
| Higher in fibre | | | | | | □ | |  | | | | | | |
| Lower in salt | | | | | | □ | |  | | | | | | |
| Not sure | | | | | | □ | |  | | | | | | |

| **Section 3: The next few items are about choosing foods** | | | | | | | | |
| --- | --- | --- | --- | --- | --- | --- | --- | --- |
|  | | | | | | | | |
| 1. | | | If a person wanted to buy a yogurt at the supermarket, which would have the least sugar/sweetener? (tick one) | | | | | |
| 0% fat cherry yogurt | | | | □ | |  | | |
| Natural yogurt | | | | ✓ | |  | | |
| Creamy fruit yogurt | | | | □ | |  | | |
| Not sure | | | | □ | |  | | |
|  | | |  | | | | | |
| 2. | | | If a person wanted soup in a restaurant or cafe, which one would be the lowest fat option? (tick one) | | | | | |
| Mushroom risotto soup (field mushrooms, porcini mushrooms, arborio rice, butter, cream, parsley and cracked black pepper) | | | | | | | □ |  |
| Carrot butternut and spice soup (carrot , butternut squash, sweet potato, cumin, red chillies, coriander seeds and lemon) | | | | | | | ✓ |  |
| Cream of chicken soup (British chicken, onions, carrots, celery, potatoes, garlic, sage, wheat flour, double cream) | | | | | | | □ |  |
| Not sure | | | | | | | □ |  |
|  | | | | | | | | |
| 3. | | | Which would be the healthiest and most balanced choice for a main meal in a restaurant? (tick one) | | | | | |
| Roast turkey, mashed potatoes and vegetables | | | | | | | ✓ |  |
| Beef, Yorkshire pudding and roast potatoes | | | | | | | □ |  |
| Fish and chips served with peas and tartar sauce | | | | | | | □ |  |
| Not sure | | | | | | | □ |  |
|  | | | | | | | | |
| 4. | | | Which would be the healthiest and most balanced sandwich lunch? (tick one) | | | | | |
| Ham sandwich + fruit + blueberry muffin + fruit juice | | | | | | | □ |  |
| Tuna salad sandwich + fruit + low fat yogurt + water | | | | | | | ✓ |  |
| Egg salad sandwich + crisps + low fat yogurt + water | | | | | | | □ |  |
| Not sure | | | | | | | □ |  |
|  | | | | | | | | |
| 5. | | | Which of these foods would be the healthiest choice for a pudding? (tick one) | | | | | |
| Berry sorbet | | | | | | | ✓ |  |
| Apple and blackberry pie | | | | | | | □ |  |
| Lemon cheesecake | | | | | | | □ |  |
| Carrot cake with cream cheese topping | | | | | | | □ |  |
| Not sure | | | | | | | □ |  |
|  | | | | | | | | |
| 6. | | | Which of these combinations of vegetables in a salad would give the greatest variety of vitamins and antioxidants? (tick one) | | | | | |
| Lettuce, green peppers and cabbage | | | | □ |  | | | |
| Broccoli, carrot and tomatoes | | | | ✓ |  | | | |
| Red peppers, tomatoes and lettuce | | | | □ |  | | | |
| Not sure | | | | □ |  | | | |
|  | | | | | | | | |
| 7. | | If a person wanted to reduce the amount of fat in their diet, but didn’t want to give up chips, which of the following foods would be the best choice? (tick one) | | | | | | |
| Thick cut chips | | | | ✓ |  | | | |
| Thin cut chips | | | | □ |  | | | |
| Crinkle cut chips | | | | □ |  | | | |
| Not sure | | | | □ |  | | | |
|  | | | | | | | | |
| 8. | One healthy way to add flavour to food without adding extra fat or salt is to add: (tick one) | | | | | | | |
| Coconut milk | | | | □ |  | | | |
| Herbs | | | | ✓ |  | | | |
| Soya sauce | | | | □ |  | | | |
| Not sure | | | | □ |  | | | |
|  | | | | | | | | |
| 9. | Which of the following cooking methods requires fat to be added? (tick one) | | | | | | | |
| Grilling | | | | □ |  | | | |
| Steaming | | | | □ |  | | | |
| Baking | | | | □ |  | | | |
| Sautéing | | | | ✓ |  | | | |
| Not sure | | | | □ |  | | | |
|  | | | | | | | | |
| 10. | Traffic lights are often used on nutrition labelling, what would amber mean for the fat content of a food? (tick one) | | | | | | | |
| Low fat | | | | □ |  | | | |
| Medium fat | | | | ✓ |  | | | |
| High in fat | | | | □ |  | | | |
| Not sure | | | | □ |  | | | |
|  |  | | | | | | | |
| 11. | “Light” foods (or Diet foods) are always good options because they are low in calories.  (tick one) | | | | | | | |
| Agree | | | | □ |  | | | |
| Disagree | | | | ✓ |  | | | |
| Not sure | | | | □ |  | | | |

| The following questions are related to food labels: | | | | |
| --- | --- | --- | --- | --- |
| 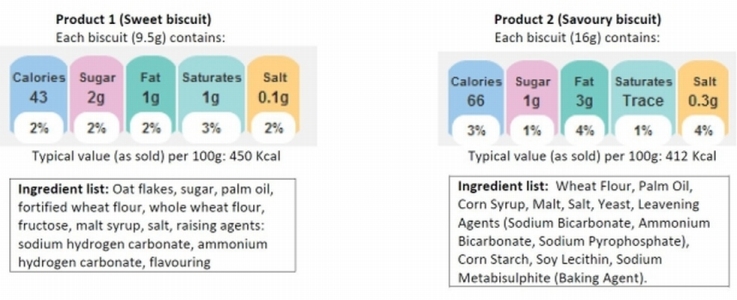 | | | | |
| 12. | | Looking at products 1 and 2, which one has the most calories (kcal) per 100 grams (tick one) | | |
| Product 1 | | | ✓ |  |
| Product 2 | | | □ |  |
| Both have the same quantity | | | □ |  |
| Not sure | | | □ |  |
|  | | | | |
| 13. | Looking at product 1, what are the sources of sugar in the ingredient list? (tick one) | | | |
| Sugar and malt syrup | | | □ |  |
| Sugar, fructose and lecithin | | | □ |  |
| Sugar, fructose and malt syrup | | | ✓ |  |
| Not sure | | | □ |  |

| **Section 4: This section is about health problems or diseases related to diet and weight management** | | | | | | |
| --- | --- | --- | --- | --- | --- | --- |
|  | | | | | | |
| 1. | | | Which of these diseases is related to a low intake of fibre? (tick one) | | | |
| Bowel disorders | | | | ✓ |  | |
| Anaemia | | | | □ |  | |
| Tooth decay | | | | □ |  | |
| Not sure | | | | □ |  | |
|  | | | | | | |
| 2. | | Which of these diseases is related to how much sugar people eat? (tick one) | | | | |
| High blood pressure | | | | □ |  | |
| Tooth decay | | | | ✓ |  | |
| Anaemia | | | | □ |  | |
| Not sure | | | | □ |  | |
|  | | | | | | |
| 3. | | Which of these diseases is related to how much salt (or sodium) people eat? (tick one) | | | | |
| Hypothyroidism | | | | □ |  | |
| Diabetes | | | | □ |  | |
| High blood pressure | | | | ✓ |  | |
| Not sure | | | | □ |  | |
|  | | | | | | |
| 4. | Which of these options do experts recommend to reduce the chances of getting cancer?  (tick one) | | | | | |
| Drinking alcohol regularly | | | | □ |  | |
| Eating less red meat | | | | ✓ |  | |
| Avoiding additives in food | | | | □ |  | |
| Not sure | | | | □ |  | |
|  | | | | | | |
| 5. | Which of these options do experts recommend to prevent heart disease? (tick one) | | | | | |
| Taking nutritional supplements | | | | □ |  | |
| Eating less oily fish | | | | □ |  | |
| Eating less trans-fats | | | | ✓ |  | |
| Not sure | | | | □ |  | |
|  | | | | | | |
| 6. | Which of these options do experts recommend to prevent diabetes? (tick one) | | | | | |
| Eating less refined foods | | | | ✓ |  | |
| Drinking more fruit juice | | | | □ |  | |
| Eating more processed meat | | | | □ |  | |
| Not sure | | | | □ |  | |
|  | | | | | | |
| 7. | | | Which one of these foods is more likely to raise people’s blood cholesterol? (tick one) | | | |
| Eggs | | | | □ | |  |
| Vegetable oils | | | | □ | |  |
| Animal fat | | | | ✓ | |  |
| Not sure | | | | □ | |  |
|  | | | | | | |
| 8. | | | Which one of these foods is classified as having a high Glycaemic Index (Glycaemic Index is a measure of the impact of a food on blood sugar levels, thus a high Glycaemic Index means a greater rise in blood sugar after eating)? (tick one) | | | |
| Wholegrain cereals | | | | □ | |  |
| white bread | | | | ✓ | |  |
| Fruit and vegetables | | | | □ | |  |
| Not sure | | | | □ | |  |
|  | | | | | | |
| 9. | | | To maintain a healthy weight people should cut fat out completely. (tick one) | | | |
| Agree | | | | □ | |  |
| Disagree | | | | ✓ | |  |
| Not sure | | | | □ | |  |
|  | | | | | | |
| 10. | | | To maintain a healthy weight people should eat a high protein diet. (tick one) | | | |
| Agree | | | | □ | |  |
| Disagree | | | | ✓ | |  |
| Not sure | | | | □ | |  |
|  | | | | | | |
| 11. | | | Eating bread always causes weight gain. (tick one) | | | |
| Agree | | | | □ | |  |
| Disagree | | | | ✓ | |  |
| Not Sure | | | | □ | |  |
|  | | | | | | |
| 12. | | | Fibre can decrease the chances of gaining weight. (tick one) | | | |
| Agree | | | | ✓ | |  |
| Disagree | | | | □ | |  |
| Not sure | | | | □ | |  |
|  | | | | | | |

| 13. | Which of these options can help people to maintain a healthy weight? (answer each one) | | | | | | | | | |  |
| --- | --- | --- | --- | --- | --- | --- | --- | --- | --- | --- | --- |
|  | | | | Yes | | | No | | Not sure |  |  |
| Not eating while watching TV | | | | ✓ | | | □ | | □ |  |  |
| Reading food labels | | | | ✓ | | | □ | | □ |  |  |
| Taking nutritional supplements | | | | □ | | | ✓ | | □ |  |  |
| Monitoring their eating | | | | ✓ | | | □ | | □ |  |  |
| Monitoring their weight | | | | ✓ | | | □ | | □ |  |  |
| Grazing throughout the day | | | | □ | | | ✓ | | □ |  |  |
|  | | | | | | | | | | |  |
| 14. | If someone has a Body Mass Index (BMI) of 23kg/m^2^, what would their weight status be?  (tick one) | | | | | | | | | |  |
| Underweight | | | | □ | | |  | | | |  |
| Normal weight | | | | ✓ | | |  | | | |  |
| Overweight | | | | □ | | |  | | | |  |
| Obese | | | | □ | | |  | | | |  |
| Not sure | | | | □ | | |  | | | |  |
|  | | | | | | | | | | |  |
| 15. | | If someone has a Body Mass Index (BMI) of 31kg/m^2^, what would their weight status be?  (tick one) | | | | | | | | |  |
| Underweight | | | | □ | | |  | | | |  |
| Normal weight | | | | □ | | |  | | | |  |
| Overweight | | | | □ | | |  | | | |  |
| Obese | | | | ✓ | | |  | | | |  |
| Not sure | | | | □ | | |  | | | |  |
| Look at the body shapes below: | | | | | | | | | | |  |
| 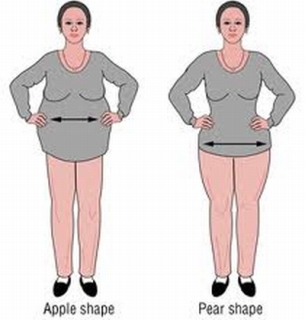 | | | | | | | | | | |  |
| 16. | Which of these body shapes increases the risk of cardiovascular disease (Cardiovascular disease is a general term that describes a disease of the heart of blood vessels, for example, angina, heart attack, heart failure, congenital heart disease and stroke)? (tick one) | | | | | | | | | |  |
| Apple shape | | | | ✓ | | |  | | | |  |
| Pear shape | | | | □ | | |  | | | |  |
| Not sure | | | | □ | | |  | | | |  |
|  | | | | | | | | | | |  |
| **Section 5: We would like to ask you a few questions about yourself** | | | | | | | | | | |  |
|  | | | | | | | | | | |  |
| 1. Are you… | | | | | | | | | | |  |
| Male | | | | | □ |  | | | | |  |
| Female | | | | | □ |  | | | | |  |
|  | | | | | | | | | | |  |
| 2. What is your current weight approximately? Please give this in stones and pounds or kilograms. | | | | | | | | | | |  |
| Stones | | |  | | | | |  | | |  |
| Pounds | | |  | | | | |  | | |  |
| Or Kilograms | | |  | | | | |  | | |  |
|  | | | | | | | | | | |  |
| 3. What is your current height approximately? Please give this in feet and inches or centimetres. | | | | | | | | | | |  |
| Feet | | |  | | | | |  | | |  |
| Inches | | |  | | | | |  | | |  |
| Or Centimetres | | |  | | | | |  | | |  |
|  | | | | | | | | | | |  |
| 4. In general, would you say your health is… | | | | | | | | | | |  |
| Poor | | | | | □ |  | | | | |  |
| Fair | | | | | □ |  | | | | |  |
| Good | | | | | □ |  | | | | |  |
| Very good | | | | | □ |  | | | | |  |
| Excellent | | | | | □ |  | | | | |  |
|  | | | | | | | | | | |  |
| 5. Are you… | | | | | | | | | | |  |
| Single | | | | | □ |  | | | | |  |
| Married | | | | | □ |  | | | | |  |
| Living as married | | | | | □ |  | | | | |  |
| Separated | | | | | □ |  | | | | |  |
| Divorced | | | | | □ |  | | | | |  |
| Widowed | | | | | □ |  | | | | |  |
|  | | | | | | | | | | |  |
| 6. Do you have any children? | | | | | | | | | | |  |
| No | | | | | □ |  | | | | |  |
| 1 | | | | | □ |  | | | | |  |
| 2 | | | | | □ |  | | | | |  |
| 3 | | | | | □ |  | | | | |  |
| 4 | | | | | □ |  | | | | |  |
| More than 4 | | | | | □ |  | | | | |  |
|  | | | | | | | | | | |  |
| 7. Do you have any children, under 18 years, living with you? | | | | | | | | | | |  |
| Yes | | | | | □ |  | | | | |  |
| No | | | | | □ |  | | | | |  |
|  | | | | | | | | | | |  |
| 8. What best describe your ethnic origin? (tick one) | | | | | | | | | | |  |
| White British | | | | | □ |  | | | | |  |
| White Irish | | | | | □ |  | | | | |  |
| Other White background | | | | | □ |  | | | | |  |
| Black British | | | | | □ |  | | | | |  |
| Black Caribbean | | | | | □ |  | | | | |  |
| Black African | | | | | □ |  | | | | |  |
| Other Black background | | | | | □ |  | | | | |  |
| Indian | | | | | □ |  | | | | |  |
| Pakistani | | | | | □ |  | | | | |  |
| Bangladeshi | | | | | □ |  | | | | |  |
| Chinese | | | | | □ |  | | | | |  |
| Other Asian background | | | | | □ |  | | | | |  |
| White and Black Caribbean | | | | | □ |  | | | | |  |
| White and Black African | | | | | □ |  | | | | |  |
| White and Asian | | | | | □ |  | | | | |  |
| Other mixed background | | | | | □ |  | | | | |  |
| Other, please specify: | | | | | | | | | | |  |
| _______________________________________________ | | | | | | | | | | |  |
|  | | | | | | | | | | |  |
| 9. What is the highest level of education you have completed? | | | | | | | | | | |  |
| Primary school | | | | | □ |  | | | | |  |
| Secondary school | | | | | □ |  | | | | |  |
| O level/ GSCEs | | | | | □ |  | | | | |  |
| A levels | | | | | □ |  | | | | |  |
| Technical or trade certificate | | | | | □ |  | | | | |  |
| Diploma | | | | | □ |  | | | | |  |
| Degree | | | | | □ |  | | | | |  |
| Post-graduate degree | | | | | □ |  | | | | |  |
|  | | | | | | | | | | |  |
|  | | | | | | | | | | |  |
| 10. Do you have any nutrition related qualifications (or are you studying to get a nutrition qualification)? | | | | | | | | | | |  |
| Yes | | | | | □ |  | | | | |  |
| No | | | | | □ |  | | | | |  |
| Please specify: | | | | | | | | | | |  |
| _______________________________________________ | | | | | | | | | | |  |
|  | | | | | | | | | | |  |
| **Thank you very much for taking part in this survey!** | | | | | | | | | | |  |
