# Supplementary material for: Reliability and validity of a revised version of the General Nutrition Knowledge Questionnaire
Source: Eur J Clin Nutr. 2016 Jun 1;70(10):1174–80. doi: 10.1038/ejcn.2016.87 (PMC5014128; doi:10.1038/ejcn.2016.87)
Supplement: Supplementary Table 2 [file ejcn201687x2.docx]

| **Changes to the General Nutrition Questionnaire** |
| --- |
| **Section 1: Dietary recommendations** |
| - Items were reworded, to make them consistent with the language used in most dietary guidelines, e.g.‘high fibre food’ was changed to ‘wholegrain foods’. - A ‘trans-fat’ option was added to the question about types of fat ^(1-4)^. - A new question covering oily fish intake was added, since it is thought to prevent Coronary Heart Disease (CHD) when consumed up to two times a week^(5, 6)^. - A new question was added about the recommendation to restrict fruit juice consumption to no more than one glass per day, due to its low fibre and high sugar content^(3, 7)^. - Questions on alcoholic drinks, breakfast and starchy food consumption were added, in line with dietary guidelines ^(6, 8, 9)^. |
| **Section 2: Sources of nutrients in food** |
| - Questions on total fat content were removed and just items covering sources of monounsaturated, polyunsaturated, saturates and cholesterol separately were retained. - A question on sources of trans-fat was added. - A question regarding the nutrient profile of processed foods and the adverse effect on human health was added, due to an increase in consumption of these foods ^(10, 11)^. - Questions about the main sources of sugar, salt, fibre and starchy foods were updated taking into account the actual major sources of these nutrients in the UK diet^(12, 13)^. - The question on which sources are low or high in protein was taken out since the specific sources of protein are more relevant than the quantity^(1, 7)^. |
| **Section 3: Knowledge of healthy food choices** |
| - Questions about the ‘traffic lights’ food labelling system, nutrition claims, ingredient lists and calorie content were added to this section, since increased consumption of processed food has resulted in the increased importance of food labels ^(1, 3, 8, 14)^. - Questions on food preparation methods were added as this is an area recommended to improve knowledge and skills in the population^(3, 12)^. - The questions on healthy meal choices were updated, covering snacks; soups; main meals; sandwich lunch; and pudding choices. - The question about antioxidants was swapped for a question about the best combination of vegetables in a salad to provide the greatest variety of vitamins and antioxidants^(15)^. |
| **Section 4: Associations between diet and ill-health** |
| - This section was expanded to include items on weight management knowledge. - Questions on Body Mass Index (BMI) classification, waist circumference and healthy behaviours to maintain a healthy weight were added based on evidence from the literature^(1, 3, 16)^. - Questions were added regarding the link between the consumption of highly refined carbohydrates and glycaemic response and diabetes prevention^(4, 7)^. - The questions about cancer and heart disease prevention were changed to a multiple choice format, as they were very similar to the first question on general dietary recommendations. - The questions about cancer and heart disease prevention were updated to provide both nutrient- and food-based recommendations options, responding to a trend towards providing mainly food-based recommendations^(17-19)^, which are more familiar and easy to understand. |
| **References** |
| 1. WHO. Global Strategy on Diet, Physical Activity and Health. Geneva: World Health Organization, 2004 92 4 159222 2.  2. Mozaffarian D, Katan MB, Ascherio A, Stampfer MJ, Willett WC. Medical progress - Trans fatty acids and cardiovascular disease. New Engl J Med. 2006;354(15):1601-13.  3. WHO. Diet, Nutrition and Prevention Diseases. Geneva: World Health Organization, 2003.  4. Skerrett PJ, Willett WC. Essentials of Healthy Eating: A Guide. J Midwifery Wom Heal. 2010;55(6):492-501.  5. Albert CM, Hennekens CH, O'Donnell CJ, Ajani UA, Carey VJ, Willett WC, et al. Fish consumption and risk of sudden cardiac death. Jama-J Am Med Assoc. 1998;279(1):23-8.  6. Nutrition SACo. The Nutritional Wellbeing of the British Population. London: Scientific Advisory Committee on Nutrition, 2008.  7. Willett WC, Stampfer MJ. Current evidence on healthy eating. Annual review of public health. 2013;34:77-95.  8. England PH. The Eatwell Guide: Helping you eat a healthy, balanced diet 2016 [cited 2016 March]. Available from: <https://www.gov.uk/government/uploads/system/uploads/attachment_data/file/510366/UPDATED_Eatwell-23MAR2016_England.pdf>.  9. Health UDo. UK Chief Medical Officers’ Alcohol Guidelines Review Summary of the proposed new guidelines 2016. Available from: <https://www.gov.uk/government/uploads/system/uploads/attachment_data/file/489795/summary.pdf>.  10. Monteiro CA, Levy RB, Claro RM, de Castro IRR, Cannon G. Increasing consumption of ultra-processed foods and likely impact on human health: evidence from Brazil. Public health nutrition. 2011;14(1):5-13.  11. Moubarac JC, Cargo M, Receveur O, Daniel M. Describing the Situational Contexts of Sweetened Product Consumption in a Middle Eastern Canadian Community: Application of a Mixed Method Design. Plos One. 2012;7(9).  12. WHO. Reducing salt in populations Geneva: World Health Organization, 2007.  13. Ministry of Agriculture FaFaOfNS. National Food Survey 2000. Colchester, Essex: UK Data Archive; 2004.  14. Spronk I, Kullen C, Burdon C, O'Connor H. Relationship between nutrition knowledge and dietary intake. Brit J Nutr. 2014;111(10):1713-26.  15. WHO. Fruit and vegetable promotion initiative. Geneva: World Health Organization, 2003.  16. Stubbs RJ, Lavin JH. The challenges of implementing behaviour changes that lead to sustained weight management. Nutrition bulletin. 2013;38:5-22.  17. Brasil. Dietary Guidelines for the Brazilian Population. Brasilia-DF: Ministry of Health of Brazil, 2014.  18. Australia. Eat for health: Australian Dietary Guidelines- Providing the scientific evidence for healthier Australian diets. Canberra: National Health and Medical Research Council of Australia, 2013.  19. WCRF/AIRC. Food, Nutrition, Physical Activity, and the Prevention of Cancer: a Global Perspective. Washington, DC,: World Cancer Research Fund/ American Institute for Cancer Research, 2007. |
