# Supplementary material for: Reliability and validity of a revised version of the General Nutrition Knowledge Questionnaire
Source: Eur J Clin Nutr. 2016 Jun 1;70(10):1174–80. doi: 10.1038/ejcn.2016.87 (PMC5014128; doi:10.1038/ejcn.2016.87)
Supplement: Supplementary Table 3 [file ejcn201687x3.docx]

Time expected and time spent to complete each survey

| **Study** | **Expected time**  **(Minutes)** | **Median Time Spent**  **(Minutes)** |
| --- | --- | --- |
| Study 1 | 15 | 14 |
| Study 2 | 15 | 14 |
| Study 3 | Used the samples from study 1 and 2. | |
| Study 4a | 15 | 18 |
| Study 4b | 5 | 4 |

4a=Online intervention 4b=Video intervention
